# Motor Unit Number Estimation (MUNE) Free of Electrical Stimulation or M Wave Recording: Feasibility and Challenges

**DOI:** 10.3389/fnagi.2022.799676

**Published:** 2022-02-11

**Authors:** Maoqi Chen, James Bashford, Ping Zhou

**Affiliations:** ^1^Faculty of Rehabilitation Engineering, University of Health and Rehabilitation Sciences, Qingdao, China; ^2^Department of Basic and Clinical Neuroscience, UK Dementia Research Institute, Maurice Wohl Clinical Neuroscience Institute, Institute of Psychiatry, Psychology and Neuroscience, King's College London, London, United Kingdom

**Keywords:** EMG-electromyogram, motor unit number estimate (MUNE), electrical stimulation, compound muscle action potential (CMAP), motor unit (MU)

## Introduction

The motor unit is the basic organizational and functional element for neuromuscular control. In motor neuron diseases, such as amyotrophic lateral sclerosis and spinal muscular atrophy, motor neurons progressively degenerate, resulting in a reduced number of motor units. Loss of motor units is also a major factor in development of sarcopenia (Gilmore et al., 2017). Although motor unit loss can be partially compensated by axonal branching and muscle fiber reinnervation, such compensation becomes insufficient with disease progression. As a result, patients suffer from progressive muscle weakness and loss of function. The number of motor units provides an important biomarker for diagnosing neuromuscular disease, tracking disease progression, and evaluating the effect of treatments and therapies (Olney and Lomen-Hoerth, 2000; Cudkowicz et al., 2006; Neuwirth et al., 2015). This has led to significant efforts in the past 50 years toward motor unit number estimation (MUNE).

## Overview of Existing Mune Methods

The first MUNE method was introduced by McComas et al. (1971) based on recording and measuring the compound muscle action potential (CMAP) and individual surface recorded motor unit potentials (SMUPs). The MUNE can then be calculated as dividing the CMAP by the mean SMUP of a sample of motor units:


$$\begin{array}{l} \text{MUNE}=\frac{\text{max}\;\text{CMAP}}{\text{mean}\;\text{SMUP}} \end{array}$$

A range of MUNE methods has been developed based on the above rationale, with each method having its advantages and limitations (Gooch et al., 2014; Carvalho et al., 2018). For all MUNE methods, it is necessary to apply supramaximal electrical stimulation of the motor nerve to record the maximal CMAP, which is derived from all motor units within the recording territory of the surface electrode. The essential difference among different MUNE methods lies in how the mean SMUP is estimated. In most MUNE methods, the mean SMUP is estimated from averaging a sample of approximately 10 motor units. For example, a sample of motor units can be obtained from incremental stimulation or multiple point stimulation of the motor nerve (Shefner et al., 2011). It can also be estimated from identification of F wave repeaters (Li et al., 2016), or spike-triggered averaging of surface electromyography (EMG) based on simultaneously recorded intramuscular EMG decomposition (Doherty et al., 2009). Recent development in high-density surface EMG provides another approach to MUNE, taking advantage of the spatial information extracted from an electrode array (van Dijk et al., 2008). High-density surface EMG decomposition can be used to estimate SMUPs without applying an invasive needle EMG electrode (Peng et al., 2016). Other MUNE methods also include Bayesian MUNE based on the CMAP scan (Ridall et al., 2006). The CMAP scan records electrical activity of a muscle in response to a full spectrum of transcutaneous stimulations of the motor nerve in approximately 500 fine steps (Visser and Blok, 2009).

Most of the existing MUNE methods are time-consuming and not automated. So far, two MUNE methods have attracted most attention in clinical application because they are automatic and quick to implement. One is called motor unit number index (MUNIX) developed by Nandedkar and colleagues (Nandedkar et al., 2004, 2010). MUNIX is based on a mathematical model, which involves analysis of the CMAP and surface EMG interference patterns taken from different levels of voluntary contraction. The other method is called MScanFit, recently developed by Bostock et al. and based on the CMAP scan (Bostock, 2016; Jacobsen et al., 2018a). It involves analysis of a large number of stimulus responses, using a progressively improved model, which takes into account the probabilistic nature of motor unit discharge.

An essential procedure of all the existing MUNE methods is to record and measure the CMAP with supramaximal stimulation of the motor nerve. This remains true regardless of the method used to estimate the mean SMUP or which signal processing approach is used. Although efforts have been made to reduce the number of electrical stimuli for estimating motor unit number (such as MUNIX and EMG decomposition-based MUNE, which only require measurement of the CMAP with supramaximal stimulation), to the best of our knowledge no attempt has been made toward a MUNE method free of electrical stimulation. Not only would this approach improve patient tolerance, but it would simplify the experimental setup, leading to greater accessibility.

## Stimulation-Free Mune

While reviewing the existing MUNE methods, we note that only SMUP waveform (area, amplitude) information is used. This is also the case for EMG decomposition-based MUNE approaches (i.e., those based on spike-triggered averaging and high-density surface EMG decomposition), even though information regarding the motor unit firing rate is available. Indeed, it is not difficult to understand that CMAP plays an indispensable role in MUNE if solely relying on SMUP waveform information. However, with EMG decomposition both the SMUP waveform and the temporal discharge information of the decomposed motor units can be obtained. By making rational use of such information, we argue that it is feasible to perform MUNE without any electrical stimulation.

EMG signal can be considered as a linear superposition of different motor unit action potential trains (MUAPTs). Consider the *i*th MUAPT as a random variable *s*_*i*_, EMG signal can be expressed as:


$$\begin{array}{l} \text{EMG}={\sum s}_{i}+n \end{array}$$

where *n* denotes the noise component. Due to the sparsity of MUAPT, any two MUAPTs can be considered uncorrelated, i.e.,


$$\begin{array}{l} E\left\{s_{i}\cdot s_{j}\right\}=0\;for\;i\;\neq \;j. \end{array}$$

Meanwhile, noise *n* can also be considered uncorrelated to any MUAPT, i.e.,


$$\begin{array}{l} E\left\{s_{i}\cdot n\right\}=0\;for\;any\;i. \end{array}$$

Consider the second moment of the EMG signal, we have


$$\begin{array}{l} m_{2}\left(EMG\right)=E\left\{EMG^{2}\right\}\text{= }E\left\{{\left(\underset{i}{\sum }s_{i}+n\right)}^{2}\right\}\text{=}\\ \underset{i}{\sum }E\left\{s_{i}^{2}\right\}+E\left\{n^{2}\right\}=\underset{i}{\sum }m_{2}\left(s_{i}\right)+m_{2}\left(n\right) \end{array}$$

where *m*_2_(*x*) represents the second moment of random variable *x*. The above equation indicates that the second moment of the EMG signal can be expressed as a summation of the second moments of its constituent individual MUAPTs and the noise component.

For maximum voluntary contraction (MVC), all motor units of the examined muscle are active. If the mean second moment of the MUAPTs is known, MUNE can be performed in a similar strategy to previous MUNE methods (based on CMAP and mean SMUP), i.e.,


$$\begin{array}{l} \text{MUNE}=\frac{m_{2}\left(EMG\right)-m_{2}\left(n\right)}{\text{mean}\;m_{2}\left(s\right)} \end{array}$$

where *m*_2_(*EMG*) can be calculated from surface EMG recorded during MVC and *m*_2_(*n*) can be calculated from the baseline (rest period) of the surface EMG signal. Calculation of *m*_2_(*EMG*) and *m*_2_(*n*) is straightforward. The key procedure of the proposed approach is to obtain the mean *m*_2_(*s*), which can be estimated from the MUAPTs of different motor units extracted by EMG decomposition (Holobar et al., 2009; Chen and Zhou, 2016). For each of the decomposed motor units, the second moment of its MUAPT can be calculated. The mean *m*_2_(*s*) can then be estimated by averaging the second moment of all the available MUAPTs from EMG decomposition.

## Challenges

Most of the MUNE methods use the rationale of maximum CMAP divided by mean motor unit size. Therefore, the accuracy is reliant on a representative sample of motor units. This imposes a major challenge for MUNE development, especially given a large range of motor unit size distribution in a muscle. The strategy of the proposed method is similar to previous CMAP-based MUNE methods in the way that it divides the second moment of maximum voluntary surface EMG by the mean second moment of the MUAPTs of a sample of individual motor units (obtained from EMG decomposition). The main difference is to replace CMAP with maximum voluntary surface EMG, and to replace SMUP area (or amplitude) with the second moment of the MUAPT. The challenge of the proposed method will also be similar to previous CMAP-based MUNE, that is, how to obtain the mean second moment of MUAPTs representative of the examined muscle? This determines the reliability of the MUNE.

For EMG decomposition, the decomposed motor units tend to have a relatively large amplitude (compared to those that cannot be decomposed in the superimposed EMG signal). It was reported that the EMG decomposition-based MUNE tends to have lower values using motor unit samples from decomposition of EMG signals at relatively high muscle contraction levels (Doherty et al., 2009). For example, in a previous EMG decomposition-based MUNE study involving M wave and voluntary surface EMG recordings (Peng et al., 2016), the estimated motor unit number with the mean motor unit size derived from 10% MVC was nearly 2–3 times the number derived from 20 and 30% MVC. A similar situation is expected for the proposed method. The mean second moment of the MUAPTs tends to be large for motor units sampled from relatively high muscle contraction levels. This will cause an underestimation of motor unit number. Because of this, it is more favorable to perform EMG decomposition at different contraction levels to obtain a less biased sample of motor units than from a single muscle contraction level. It is also helpful to increase the decomposition yield (i.e., extracting a larger number of motor units) to have a more representative motor unit sample. It is worth noting that for previous EMG decomposition-based MUNE, for each motor unit a partial decomposition would be sufficient to obtain SMUP waveform template. For the proposed method, a complete decomposition is required for decomposed motor units to calculate the second moment of MUAPTs.

## Concluding Remarks

By proposing a novel strategy to perform MUNE without recording the CMAP, we argue that stimulation-free MUNE is feasible. The challenge remains in the acquisition of representative motor unit samples for calculation of the mean second moment of MUAPTs, which is critical for the performance of the proposed MUNE method. The performance is also affected by other complex neurophysiological factors such as motor unit distribution and the level of motor unit synchronization, and thus requires further investigation. Our future work will involve both surface EMG simulation and experimental approaches for evaluating the performance of the stimulation-free MUNE approach, in terms of diagnostic accuracy or sensitivity, as compared with CMAP-based MUNE methods (Blok et al., 2005; Major and Jones, 2005; Boekestein et al., 2012; Li et al., 2012; Jacobsen et al., 2018b). Since there is a lack of 'ground truth' regarding the real motor unit number in human subjects, it is difficult to experimentally quantify a MUNE method's accuracy in terms of absolute motor unit number (especially given the uncertainty of the muscle volume a surface electrode can record from). Therefore, like various existing MUNE methods, such investigation should focus on the sensitivity of following disease progression when serial investigations are performed, rather than the absolute numerical result. If the stimulation-free MUNE proves to be reasonably sensitive to motor unit number changes, it provides a novel biomarker for monitoring disease progression and holds value for clinical trials. Importantly, the advent of a stimulation-free approach would facilitate home-based MUNE assessments, leading to notable practical and analytical advantages.

## Author Contributions

MC wrote the first draft. JB and PZ revised the manuscript. All authors contributed to the conception and design of the work and approved the submitted version.

## Funding

The study was supported by the Shandong Provincial Natural Science Foundation under grant nos. ZR2021QH053 and ZR2020KF012. JB was supported by a National Institute for Health Research Clinical Lectureship in Neurology.

## Conflict of Interest

The authors declare that the research was conducted in the absence of any commercial or financial relationships that could be construed as a potential conflict of interest.

## Publisher's Note

All claims expressed in this article are solely those of the authors and do not necessarily represent those of their affiliated organizations, or those of the publisher, the editors and the reviewers. Any product that may be evaluated in this article, or claim that may be made by its manufacturer, is not guaranteed or endorsed by the publisher.
